# *In vivo *whole-cell patch-clamp recording of sensory synaptic responses of cingulate pyramidal neurons to noxious mechanical stimuli in adult mice

**DOI:** 10.1186/1744-8069-6-62

**Published:** 2010-09-28

**Authors:** Kohei Koga, Xiangyao Li, Tao Chen, Hendrik W Steenland, Giannina Descalzi, Min Zhuo

**Affiliations:** 1 Department of Physiology, Faculty of Medicine, University of Toronto, Medical Science Building, 1 King's College Circle, Toronto, Ontario M5S 1A8, Canada; 2 Department of Brain and Cognitive Sciences, Seoul National University, Seoul 151-746, Korea

## Abstract

The anterior cingulate cortex (ACC) plays important roles in emotion, learning, memory and persistent pain. Our previous *in vitro *studies have demonstrated that pyramidal neurons in layer II/III of the adult mouse ACC can be characterized into three types: regular spiking (RS), intermediate (IM) and intrinsic bursting (IB) cells, according to their action potential (AP) firing patterns. However, no *in vivo *information is available for the intrinsic properties and sensory responses of ACC neurons of adult mice. Here, we performed *in vivo *whole-cell patch-clamp recordings from pyramidal neurons in adult mice ACC under urethane anesthetized conditions. First, we classified the intrinsic properties and analyzed their slow oscillations. The population ratios of RS, IM and IB cells were 10, 62 and 28%, respectively. The mean spontaneous APs frequency of IB cells was significantly greater than those of RS and IM cells, while the slow oscillations were similar among ACC neurons. Peripheral noxious pinch stimuli induced evoked spike responses in all three types of ACC neurons. Interestingly, IB cells showed significantly greater firing frequencies than RS and IM cells. In contrast, non-noxious brush did not induce any significant response. Our studies provide the first *in vivo *characterization of ACC neurons in adult mice, and demonstrate that ACC neurons are indeed nociceptive. These findings support the critical roles of ACC in nociception, from mice to humans.

## Background

Recent reports from animals and human imaging studies demonstrate that the anterior cingulate cortex (ACC) plays important roles in many major physiological functions as well as pathological conditions [1-8]. A number of human brain imaging studies have shown that ACC activity is involved in different types of pain-related information processing [9-14]. To understand the basic cellular and molecular mechanisms of the ACC, *in vitro *electrophysiological studies of ACC cortical slices and the use of genetically manipulated mice have provided critical information about excitatory transmission and long-term plastic changes after peripheral tissue or nerve injuries [7,8,15-21]. These findings from normal and transgenic/knockout mice are in good accord with *in vivo *electrophysiological studies in rats and rabbits [5,22]. However, fewer studies of nociceptive responses from ACC neurons have been reported from whole adult mice due to the technical limitations involved in *in vivo *electrophysiological recordings. Considering the increases of *in vitro *studies and the use of transgenic and gene knockout mice for the molecular study of ACC functions, it is necessary to study the intrinsic properties of ACC neurons under whole mouse preparations. Of particular interest is how the ACC is activated by *in vivo *sensory stimulation. To our knowledge, there has been no report of *in vivo *intrinsic electrophysiological properties of ACC neurons.

Here we report for the first time *in vivo *whole-cell patch-clamp recordings from the ACC of adult mice. Our unique *in vivo *whole-cell patch-clamp approach has the following major technical advantages: (1) the analyses of membrane potentials and synaptic currents of spontaneous excitatory postsynaptic currents (sEPSCs); (2) recording of evoked synaptic responses (EPSCs) induced by different types of peripheral sensory stimuli, (3) a long stable recording (up to 2 hrs) and (4) anatomic labeling of recorded neurons. We found that many ACC neurons are nociceptive, and show selective responses to peripheral noxious mechanical stimuli. Furthermore, ACC neurons showed bilateral responses to noxious (pinch) stimuli applied to both ipsilateral and contralateral hind paws.

## Results

### *In vivo *recording from layer II/III pyramidal neurons of ACC

**Figure 1:**
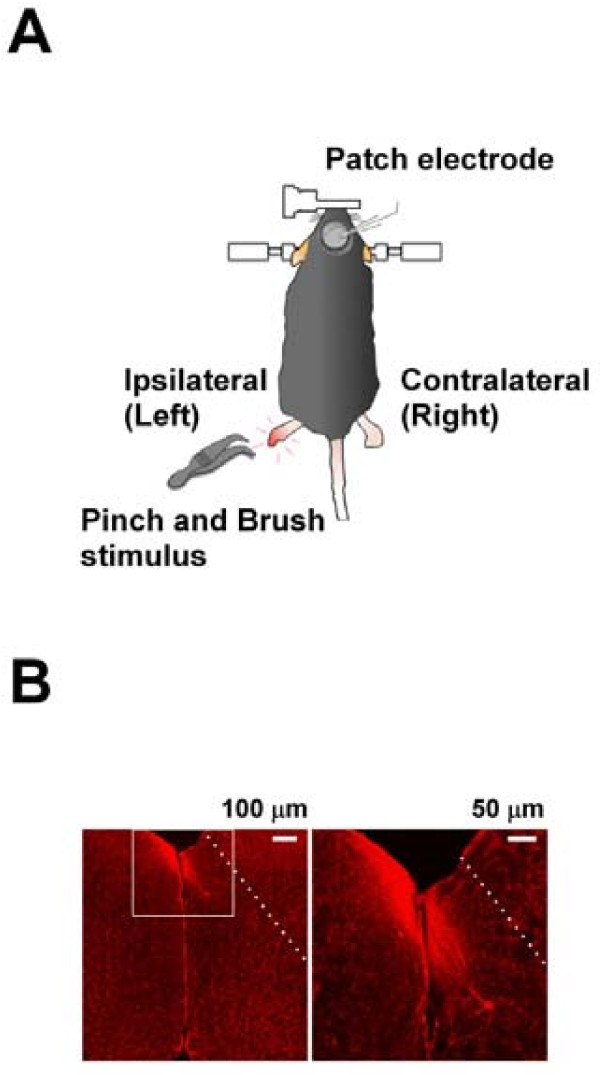
**Schematic diagram and the identification of the recording location**. **(A) **Schematic diagram showing the *in vivo *preparation. **(B) **The identification of the recording location. The photomicrograph of a representative biocytin-labeled layer II/III ACC pyramidal neuron as visualized with confocal laser scanning microscopy.

To characterize ACC neurons of adult mice *in vivo*, we carried out whole-cell patch-clamp recordings from neurons in the superficial layers of adult mouse ACC under anesthesia (Fig 1A). ACC neurons were electrophysiologically characterized and stained with biocytin at the end of the experiments (Fig 1B). Unlike *in vitro *slice recordings, the success rate for forming high seal resistances of more than 5 GΩ was about 20% of all trials. The membrane patch was ruptured by applying an additional negative pressure to obtain the whole-cell recording configuration. Typical recording durations were about 30 min or more. One to three cells could be thoroughly investigated each day. In the present study, a total of fifty pyramidal neurons in the ACC were obtained from 31 adult mice.

### Neuronal firing patterns and slow oscillations

**Figure 2:**
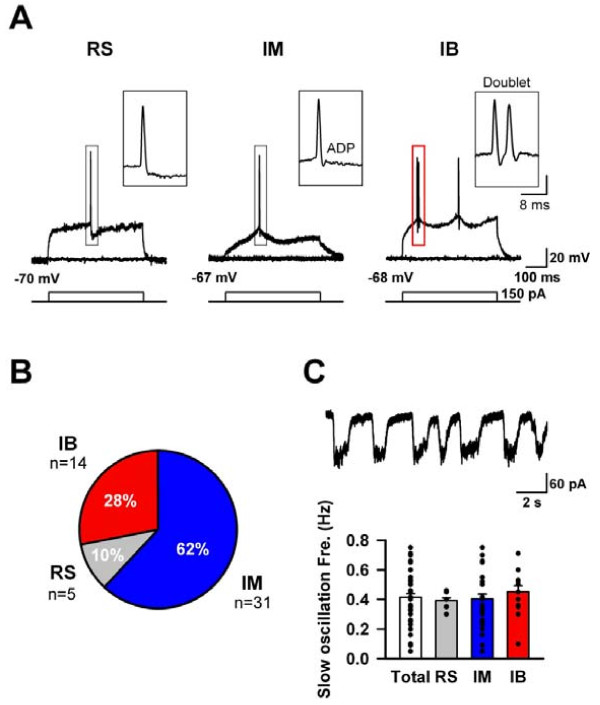
**Intrinsic membrane properties of pyramidal neurons *in vivo***. **(A) **Characteristics of the typical firing patterns in RS (left), IM (middle) and IB (right) evoked by current injections (300 ms, 50 pA step) at resting membrane potentials. These types of neurons exhibited slow afterhyperpolarization (sAHP; ms, mV) with a delayed slow component. Note that the IB neuron has the AP doublet. **(B) **The population ratios of different neuron groups for RS, IM and IB. **(C) **Voltage clamp recording at -70 mV. There was no significant difference in the slow oscillations of each type.

Recently, using *in vitro *cortical slice preparations we have demonstrated that pyramidal neurons in layer II/III of the ACC can be divided into three major different types according to their firing patterns and the shapes of their action potentials (APs) [16,23]. However, it is unclear if these patterns obtained from *in vitro *slices reflect *in vivo *conditions. In the present *in vivo *study, we made similar observations. ACC pyramidal neurons were classified into three categories as reported previously from *in vitro *brain slices: (i) regular spiking (RS) cells, in which the single spike is followed by a slow afterhyperpolarization (AHP) (Fig. 2A left); (ii) intermediate (IM) cells, in which the single spike is followed by a fast AHP and a fast afterdepolariztion (ADP) (Fig. 2A middle); and (iii) intrinsic bursting (IB) cells that in particular fired an initial spike doublet (Fig. 2A right). Fig. 2B shows the percentage ratios of individual types obtained from the present *in vivo *study. By far the most frequently recorded type was IM cells (n = 31, 62%), whereas only 5 out of 50 pyramidal neurons were classified as RS (10%), and only 14 out of 50 recorded neurons were IB cells (28%). Interestingly, in contrast to the populations of RS (25%) and IM (44%) cells described in *in vitro *brain slices of adult mice [16], the RS (10%) and IM ratios (62%) were significantly different under *in vivo *conditions (*P *< 0.01, χ^2 ^test).

We also characterized the general intrinsic properties of all three types of ACC cells (Table. 1). Our analysis revealed an average resting membrane potential of -69.0 ± 1.6 mV, an average spontaneous AP threshold (V_Threshold_) of -42.3 ± 1.8 mV, an AP amplitude of 58.9 ± 4.3 mV on V_Threshold _and a half width of 1.4 ± 0.2 ms. There was no significant difference among different cell types. However, the values of the ADPs, which are considered to indicate a tendency for a neuron to fire in bursts, in IB (13.1 ± 3.3 mV) were significantly higher than those in RS and IM (9.7 ± 1.3 mV) (Fig. 2A and Table. 1).

**Table 1:** Summary of basic electrophysiological parameters of ACC layer II/III pyramidal neurons in adult mice *in vivo*

	*RS cells (n = 5)*	*IM cells (n = 31)*	*IB cells (n = 14)*
***Ratio***	10	62	28
***RMP (mV)***	-68.4 ± 2.2	-72.0 ± 1.9	-67.1 ± 2.3
***Spontaneous AP threshold (mV)***	-46.1 ± 2.9	-48.3 ± 1.4	-44.2 ± 2.0
***Oscillation Frequency (Hz)***	0.4 ± 0.08	0.41 ± 0.04	0.48 ± 0.04
***Spike Height (mV)***	56.1 ± 7.5	59.0 ± 2.3	57.5 ± 3.2
***Spike Half Width (ms)***	1.40 ± 0.23	1.40 ± 0.08	1.37 ± 0.09
***fAHP Amplitude (mV)***	ND	-7.1 ± 1.4	-7.1 ± 2.2
***Time of fAHP (ms)***	ND	2.2 ± 0.1	2.1 ± 0.2
***sAHP Amplitude (mV)***	-10.5 ± 2.0	-10.3 ± 0.8	-9.2 ± 1.6
***Time of sAHP (ms)***	65.8 ± 11.4	68.5 ± 9.4	59.8 ± 8.3
***ADP (mV)***	ND	9.7 ± 1.3^a^	13.1 ± 3.3^a, b^
***Pinch-evoked responses higher than 200%***	1/5 (20%)	10/31 (32%)	7/14 (50%)

Values are means ± SEM.

*n *is number of cells.

ACC, anterior cingulate cortex; RMP, resting membrane potential; fAHP, fast afterhyperpolarization; sAHP, slow afterhyperpolarization; ADP, afterdepolarization.

^a ^Significantly different from RS cells at *P *< 0.001.

ND indicates no data.

^b ^Significantly different from IM cells at *P *< 0.05.

In this study, we used urethane for animal's anesthesia *in vivo*. Urethane anesthesia is known to affect membrane potentials and produce slow oscillations which have up- and down-states [24-26]. To confirm that the classifications of firing patterns were not affected by different depths of urethane anesthesia, we next analyzed slow oscillations in individual types of cells. Although space clamp errors under voltage clamp conditions should be taken into consideration, the recording conditions under *in vivo *patch-clamp allow us to directly analyze the synaptic currents. Similar direct analyses have been successfully implemented in the spinal cord [27,28] and primary somatosensory cortex [24]. The voltage clamp recordings revealed inward slow oscillations of membrane currents at the holding membrane potential (VH) of -70 mV (Fig. 2C upper). The mean frequency of the oscillations was 0.42 ± 0.02 Hz, ranging from 0.05 to 0.75 Hz (n = 50) and were similar as in other cortical area [24,25]. The spontaneous oscillation frequencies of individual types were similar in RS (0.40 ± 0.08 Hz), IM (0.41 ± 0.04 Hz) and IB cells (0.48 ± 0.04 Hz) (Fig. 2C bottom).

### Spontaneous APs in response to innocuous brush in all cell types

**Figure 3:**
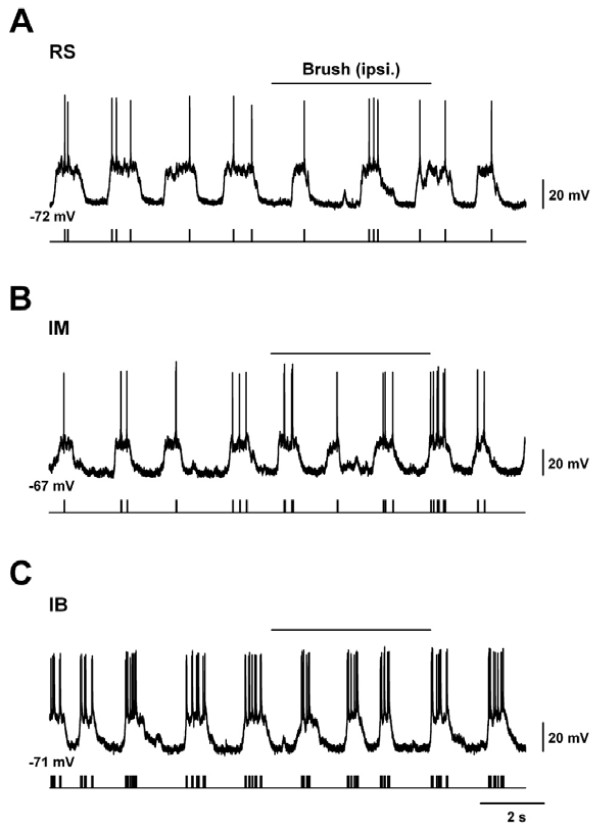
**Spontaneous AP frequencies and responses to peripheral innocuous brush stimuli in three different types of ACC neurons**. **(A-C) **Spontaneous firing and responses to 'innocuous' brush stimuli *in vivo *in RS (A), IM (B) and IB (C) under current clamp condition. Raster plots of spontaneous and evoked action potentials (A-C bottom).

**Figure 4:**
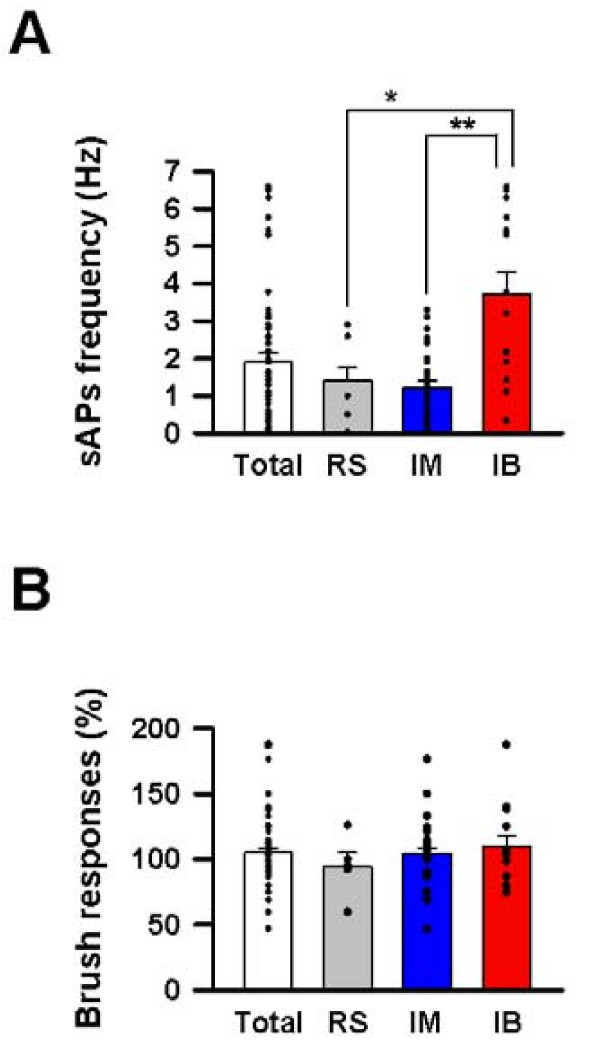
**Comparisons of spontaneous AP frequencies and innocuous brush stimuli in three different types of ACC neurons**. **(A) **Spontaneous firing frequency in IB increased compared with those in RS and IM (*P < 0.05, ** *P *< 0.01). **(B) **Brush did not activate all three types of cells.

We next analyzed the spontaneous firing of action potentials (sAPs) which occurred during the up state of slow oscillations (Fig. 3, 4) [24-26]. Specifically, we assessed the sAPs frequencies on the up states of membrane potentials in the ACC. The average sAP frequency in RS and IM cells were 1.4 ± 0.3 Hz (Fig. 3A, 4A) and 1.2 ± 0.2 Hz (Fig. 3B, 4A), respectively. Interestingly, the APs frequency in IB cells was 3.7 ± 0.6 Hz, which was significantly higher than those in RS and IM (Fig. 4A, *P *< 0.05 for RS, *P *< 0.01 for IM, one way ANOVA).

Although it is known that innocuous stimuli does not significantly activate neurons in the ACC [5,8], to our knowledge there is no systematic study that has examined innocuous stimulation evoked responses in individual cell types in the superficial layers of the ACC. Therefore, we first applied innocuous brush stimuli to the left hind paw. As expected, the brushing did not produce any evoked depolarizations with an AP at the onset of the stimuli in all cell types under urethane anesthesia (RS; 94.7 ± 10.5%, IM; 104.3 ± 4.1% and IB; 110.1 ± 8.0% vs control) (Fig. 3, 4B).

### Noxious pinch evoked responses

**Figure 5:**
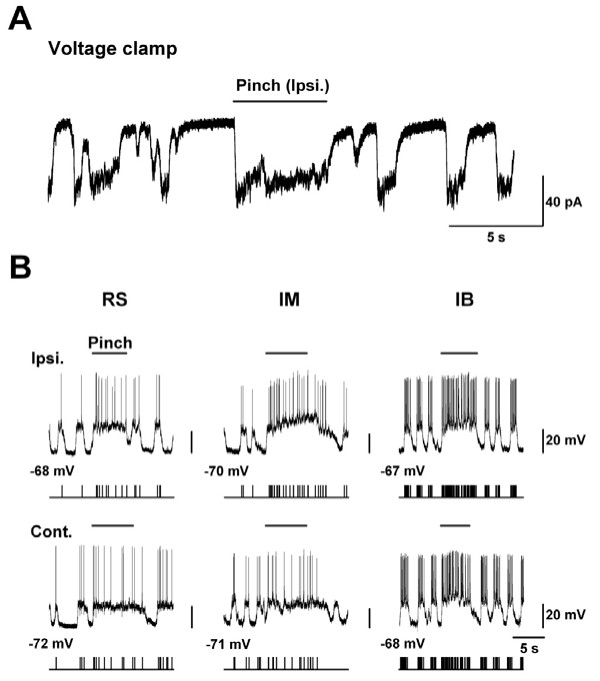
**Responses of ACC neurons to peripheral noxious mechanical pinch stimuli**. **(A) **EPSCs evoked by pinch (ipsilateral) recorded in voltage clamp at -70 mV in a typical IM cell. **(B) **Action potentials evoked by pinch stimuli applied to ipsilateral (upper) and contralateral (bottom) hind paws in RS (right), IM (middle) and IB (left). These responses were obtained in the same neuron from each type. Raster plots of spontaneous and evoked action potentials in each type.

Noxious pinch evoked responses in a cell type-specific manner within the ACC remain unknown. Therefore we next applied cutaneous noxious pinch stimuli to the hind paws of adult mice and characterized evoked responses across all cell types (Fig. 5). Under voltage-clamp conditions, pinching produced long-lasting inward membrane currents throughout the duration of stimulation, and the evoked responses disappeared immediately after the stimuli were terminated (Fig. 5A). In addition, under current clamp conditions, we observed pinch evoked membrane potentials in the same neuron in response to both ipsilateral (Fig. 5B upper) and contralateral (Fig. 5B bottom) hind paw stimulation. The pinch stimuli applied to both hind paws elicited neuronal depolarization with increasing APs frequencies in RS and IM neurons, particularly at the onset of stimulation (Fig. 5A left and middle). Interestingly, in most of the IB cells, the stimuli produced a barrage of APs frequencies throughout the stimulation period (Fig. 5B right).

**Figure 6:**
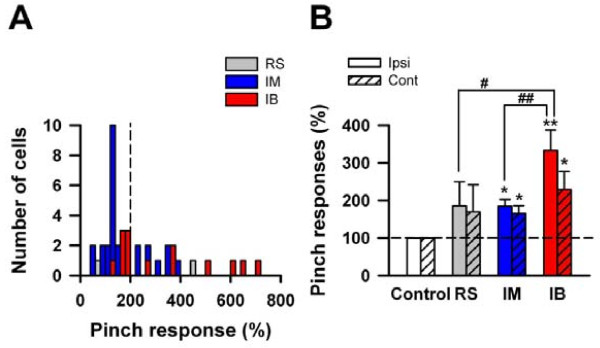
**Comparisons of the responses to peripheral noxious mechanical pinch in ACC neurons**. **(A) **The histogram of pinch-evoked responses in individual types (RS; grey, IM; blue and IB; red). **(B) **The summary of Fig. 5B. Pinch activated all types (*P < 0.05, **P < 0.01, compared with control, one way ANOVA). The pinch-evoked response in IB significantly increased, compared with those in RS and IM (#*P *< 0.05, ##*P *< 0.01, one way ANOVA). There were no changes between ipsilateral and contralateral evoked responses in all types.

Finally, we summarized pinch-evoked responses in individual cell types (Fig. 6). We considered each spontaneous AP frequency as a control (100%), and plotted the number of cells which were facilitated by pinch (Fig. 6A). We compared the percentage of cells from each group that displayed more than a 200% increase from control in AP frequency in response to pinch, and observed that the number of the cells which showed these increases were different between individual cell types. Specifically, pinch stimuli increased the response frequency of one in five RS neurons (20%), while 10 of 31 IM neurons (32%) were activated by the stimuli. In comparison, 50% of IB neurons (7/14) were excited by pinch stimuli (Fig. 6A). We further compared the ipsilateral and contralateral pinch evoked responses between individual cell types. The average frequencies of ipsilateral pinch evoked responses in RS and IM were 185 ± 65% and 185 ± 17%, respectively. In contrast, the average response frequency of IB cells was 333 ± 54%, significantly higher than those of RS and IM cells (Fig. 6B, *P *< 0.05 for RS, *P *< 0.01 for IM, one way ANOVA). Similar evoked-responses to contralateral pinch stimulation were observed in all cell types (Fig. 6B, contralateral responses in RS, IM and IB were 170 ± 72, 165 ± 21 and 229 ± 48%, respectively), suggesting that ACC neurons receive bilateral noxious input from the periphery.

## Discussion

The present study is the first to perform *in vivo *patch-clamp recordings from ACC neurons of adult mice under urethane anesthesia and to systematically characterize the action potential (AP) properties of layer II/III pyramidal neurons. From single labeled neuron morphological analyses, we confirmed recording positions. We chose to record from layer II/III neurons in the ACC because (1) our previous work mostly focused on synaptic transmission and plasticity of ACC layer II/III [18,20,21]; (2) most neurons located in layer II/III are pyramidal neurons; and (3) neurons in layer II/III receive robust sensory input. We found that three major electrophysiological classes of pyramidal neurons could be distinguished according to their firing patterns and the shapes of their APs: RS, IM and IB neurons. Finally, we applied cutaneous noxious pinch and innocuous brush stimuli to the ispilateral and/or contralateral hind paws whilst recording from each type of neuron. We revealed that the pinch-evoked responses in IB cells had significantly higher frequencies than those in RS and IM, although brush stimuli did not activate any cell type. In contrast, there were no significant differences between ispilateral and contralateral evoked-responses across all cell types.

### Firing patterns

Based on AP shape and specific discharge patterns, our current study recorded from three identified types of pyramidal neurons within the ACC, which composed 10, 62 and 28% of our recordings respectively. In contrast, using the same classification protocols, our previous *in vitro *spike study [16] demonstrated different population distributions for ACC RS, IM and IB pyramidal neurons in layer II/III of adult mice (25, 44 and 31%, respectively). In comparison with the *in vitro *study, IM neurons were more frequently observed (62% *in vivo *vs. 44% *in vitro*). On the other hand, the number of RS neurons observed under *in vivo *conditions (10%) was lower than under *in vitro *conditions (25%) although the percentage of IB (28%) neurons observed in our *in vivo *study was similar to those observed *in vitro *(31%). The population ratios of RS and IM cells under *in vivo *and *in vitro *conditions were significantly different (*P *< 0.01, χ^2 ^test). One key conclusion from these studies is that *in vitro *brain slice preparations is useful for studying the spike properties of different types of ACC pyramidal cells *in vivo*.

It is important to note that some of the variations in the population ratios between *in vivo *and *in vitro *observations may be related to recording conditions including (i) anesthetic effects and (ii) variations in recording temperatures. The generation and shape of action potentials depend on sodium (Na), potassium (K) and calcium (Ca) channels [29,30]. The main criteria distinguishing IM cells from RS cell is the presence of the afterdepolarization (ADP), which is generally composed of Na, T- and R-type Ca currents [29]. Compared with *in vitro *conditions, *in vivo *states are more likely to maintain neurons in a healthy condition, and thus these channels may be more active than under *in vitro *conditions. In addition, it has been reported that urethane can affect various channels, including Na channels [31-33]. Although there were no differences in slow oscillation frequencies produced by urethane in the three types of cells (Fig. 2C), we cannot eliminate the effects of anesthesia on firing pattern classifications. Furthermore, our previous *in vitro *study [16] was conducted at room temperature, whereas the present study used recording temperatures ranging from 36-37°C. These differences in temperature may affect channel gating, which could explain a difference in ADP amplitude and hence the proportion of cells that are RS vs IM.

### Spontaneous and evoked responses

We observed that the spontaneous APs frequencies in IB neurons were significantly higher than those in RS and IM neurons (Fig. 4A). It has been reported that the generation of AP bursting discharges are mainly dependent on Na and K channel activity [29,30]. In fact, by raising extracellular K concentrations, or through potentiation of the persistent Na current with the Na channel toxin ATX II, a subset of cortical pyramidal neurons can be induced to switch from repetitive single spiking to a burst firing mode [30]. Additionally, the blocking of M-current, which is a voltage-gated potassium current (current through Kv7 channels) and a low-threshold non-inactivating potassium current, neuronal firing can be switched from tonic discharge to an intermittent firing mode in the entorhinal cortex [34] and hippocampus [35]. Therefore, it is possible that individual types of pyramidal neurons in the ACC may have different expressions of AP related channels.

We also found that IB neurons responded significantly more to noxious pinch stimuli (333%) than RS (185%) and IM cells (185%) (Fig. 6B). A possible explanation for this may be, in addition to differences in their intrinsic properties, that they exhibit differences in sensory input and anatomical features of individual types. It has been reported that IB and RS neurons in the neocortex have different characteristics in excitatory and inhibitory input [36-38]. IB neurons mainly receive excitatory input and rarely receive GABAergic inhibitory input [38]. Furthermore, they receive smaller and slower rises and decays of GABAergic inputs than RS cells [36,37]. Anatomically, IB neurons have larger cell bodies and longer, thicker apical dendrites than those in RS cells [36-38]. The functional implications of their intrinsic electrophysiological properties, their responses to various sensory stimuli and their morphological features need to be further investigated.

### Functional implications

Neurons in layer II/III of the ACC receive sensory information from the medial thalamus, and in turn project to layer V neurons [7,8,39-41]. Layer V neurons in turn form synapses with various cortical areas including the amygdala, a structure critical for emotional fear and anxiety, and also the motor cortex, where they can generate motor responses such as emotional vocalizations or trigger aversive behaviors [4,42,43]. Taking into considerations of the different intrinsic physiological properties and pinch-evoked responses obtained in this study, it is possible that different classes of pyramidal neurons within the ACC contribute in different ways to the information processing that takes place in the ACC. In fact, when noxious stimuli was applied, RS and IM cells displayed phasic firing at the onset of the stimuli, whereas IB cells displayed a tonic firing pattern, acting like powerful amplifiers during stimulation (Fig. 5B). Additionally, noxious pinch stimuli induced increases in evoked AP frequency greater than 200% of control in 50% of IB neurons (Fig. 6A). Therefore, the generation of high-frequency burst discharges in IB neurons may strongly influence the responses of postsynaptic neurons and the operation of local cortical networks, and also may play important roles in various emotional and motor responses.

## Conclusions

Our current spike study is the first *in vivo *study in ACC neurons of adult mice, and provides basic information of the intrinsic properties and noxious responses of pyramidal ACC neurons. In conjunction with acute pain and neuropathic pain models, *in vivo *patch clamp recordings would be a powerful technique. Considering the cumulative reports of studies using transgenic and gene knockout mice, the current study provides important information of the intrinsic properties for ACC neurons. The *in vivo *whole-cell patch-clamp method will allow us to reveal novel molecular and synaptic integrative mechanisms for synaptic transmission and plasticity within the ACC in the future.

## Methods

### Animal preparations

Adult male C57BL/6 mice (8-12 weeks old) were used in this study. Anesthesia was induced with urethane (1.5 g/kg, i.p.) as described previously [24]. Rectal temperature was kept at 36-37°C by means of a heating pad placed beneath the animal. The head of the mouse was fixed with a stereotaxic apparatus, and the skull was drilled to make a hole on the right parietal hemisphere (0-0.7 mm lateral to the midline, and 1.0 mm anterior to bregma) [18]. The dura was partially removed and a plastic well with 5 mm height was fixed around the hole with an adhesive bond. The surface of the ACC was perfused with 95% O_2_-5% CO_2 _equilibrated Krebs solution at 37°C (mM: NaCl 124, KCl 2.5, CaCl_2 _2.0, MgSO_4 _2.0, NaH_2_PO_4 _1.0, NaHCO_3 _25, and glucose 10, pH 7.4, 300-310 mOsm). If mice moved, a 1/4 supplemental dose of urethane was provided. The reflex was also monitored. All experiments were performed under protocols approved by the University of Toronto Animal Care Committee.

### *In vivo *Whole-cell patch-clamp recording

Blind whole-cell patch-clamp recordings were made from ACC neurons. The recordings were obtained with a patch electrode filled with a solution (mM: K-gluconate 120, KCl 5, CaCl_2 _0.5, MgCl_2 _1, EGTA 0.2, HEPES 10, and Mg-ATP 2, Na_3_-GTP 0.1 and phosphocreatine disodium 10 adjusted to pH 7.2 with KOH). Biocytin (0.2%) was included in the pipette solution to label recorded neurons. The electrode, with a resistance of 2-5 MΩ, was advanced at an angle of 65 degrees towards the midline, into the range of the ACC (from 0.15-0.4 mm lateral to midline, and from 0.2-0.8 mm anterior to bregma, and 0.2-0.5 mm ventral from surface of the brain) [44]. After making a giga Ohm seal (the resistance of at least 5 GΩ), the membrane patch was ruptured by a brief period of negative pressure, thus resulting in the whole cell configuration. Access resistance < 50 MΩ was considered acceptable. Data were discarded if access resistance changed more than 15% during an experiment. Data were filtered at 2 kHz, and digitized at 10 kHz. A 10 mV liquid junction potential was subtracted from all membrane potentials.

### Somatosensory stimuli

Innocuous (paint brush) and noxious pinch (toothed forceps) stimuli were applied for 3-5 seconds to the skin of the left (ipsilateral) and/or right (contrarateral) hind paws. All stimuli were usually applied at intervals of at least 3 min.

### Histology and confocal microscopy

After electrophysiological recordings, mice were deeply anesthetized with supplemental urethane, and perfused transcardially with 4% paraformaldehyde in 0.01 mM phosphate-buffered saline (PBS, pH 7.4). The cortex was removed and immersed overnight in the same fixative at 4°C, and rinsed in 0.01 mM PBS and sectioned transversely into 80 μm-thick slices using a Cryostat. The biocytin filled cells were rendered fluorescent by incubating overnight in a Cy3-conjugated streptavidin (Jackson ImmunoResearch Labs; West Grove, PA) solution (1 mg/ml of PBS) at 4°C. The following day, slices were equilibrated in 1% Tris buffered saline and mounted on glass slides. Labeled neurons were imaged by a confocal microscope (Fluoview FV 1000, Olympus, Tokyo, Japan).

### Statistical analysis

Data are expressed as the mean ± SEM. Statistical significance was determined as *P < 0.05 *using the Student *t*-test, the Mann-Whitney U-test, the analysis of variance (ANOVA) or chi-square (χ^2^) test, and indicated by asterisks.

## List of abbreviations

ACC: anterior cingulate cortex; APs: Action potentials; RS: regular spiking neurons; IM: intermediate neurons; IB: intrinsic bursting neurons; RMP: resting membrane potential; fAHP: fast afterhyperpolarization; sAHP: slow afterhyperpolarization; ADP: afterdepolarization; V_H: _holding potential; V_threshold: _action potential voltage threshold;

## Competing interests

The authors declare that they have no competing interests.

## Authors' contributions

KK carried out electrophysiology and drafted the manuscript. XL participated in electrophysiological experiments. TC helped with confocal experiments. HS helped with data analysis. GD drafted the manuscript. MZ designed and finished the final draft of the manuscript. All authors read and approved the final manuscript.
